# Traumatic Events Predict Eating Disorders Among Palestinians: The Moderating Role of Demographic Variables

**DOI:** 10.1002/brb3.70460

**Published:** 2025-04-23

**Authors:** Fayez Mahamid, Bilal Hamamra, Dana Bdier

**Affiliations:** ^1^ Faculty of Humanities and Educational Sciences An‐Najah National University Nablus Palestine

**Keywords:** eating disorders, Palestine, sociodemographic variables, traumatic events

## Abstract

**Objectives:**

The current study aimed to examine the relationship between traumatic events and eating disorders (EDs), as well as the moderating effect of selected sociodemographic factors (educational level, gender, region, and age) within the Palestinian context.

**Methods:**

Participants of the current study were 580 adults, including 320 males and 260 females, who were recruited online using convenience sampling techniques.

**Findings:**

Results of the correlational analysis revealed that traumatic events positively correlated with restraint eating (*r* = 0.41*, p *< 0.01), eating concern (*r* = 0.42, *p* < 0.01), weight concern (*r* = 0.43, *p *< 0.01), shape concern (*r* = 0.46, *p *< 0.01), and EDs total (*r* = 0.40, *p *< 0.01). Results of hierarchical regression revealed that EDs were predicted by both sociodemographic variables and traumatic events (*β* = 0.44; *p < 0.01*).

**Conclusion:**

The findings open the door for further research to better understand how the variables in this study correlate with one another. This would allow for the development and implementation of effective clinical interventions aimed at reducing EDs by promoting positive coping strategies for dealing with traumatic events, with support from mental health professionals.

## Graphical abstract

***Key Findings***: • Traumatic events positively correlate with various ED symptoms. • Sociodemographic factors (gender, age, region, education) moderate these effects. • Hierarchical regression shows that both trauma and sociodemographic variables predict EDs. ***Conclusion/Implications***: Understanding trauma's role in EDs among Palestinians could lead to better‐targeted interventions based on sociodemographic factors.

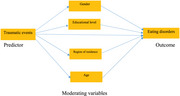

## 1 Theoretical Background

Eating disorders (EDs) represent complex conditions shaped by an interplay of biological, psychological and socio‐environmental factors. Globally, incidence of EDs has increased; scholarly research predominantly concentrates on Western populations. Studies frequently link EDs to societal pressures, media influence and psychological vulnerabilities—such as perfectionism and low self‐esteem (Treasure et al. 2020; Smink et al. 2012). However, emerging evidence highlights distinct risk factors that prevail in non‐Western and conflict‐affected regions; here, chronic stress, food insecurity and cultural stigmas converge to shape eating behaviors (Musaiger et al. 2013). Conflict zones, in particular, foster environments where disrupted daily routines, resource scarcity and psychological trauma contribute to maladaptive eating patterns. Although importance of examining EDs in diverse contexts is recognized, much of current literature remains entrenched in Western‐centric frameworks, overlooking the complexities of EDs in conflict zones.

This oversight is troubling considering that such regions frequently encounter compounded vulnerabilities that intensify the prevalence and severity of eating disorders (Jacobi et al. 2004). Addressing this gap necessitates an understanding of how prolonged instability, displacement, and cultural factors interact to influence the development of EDs. The Palestinian context presents a crucial case study because decades of the Israeli occupation and systemic oppression have engendered conditions of chronic trauma and food scarcity. This situation, however, escalates the risk of EDs among vulnerable populations (Thabet et al. 2015).

Conflict introduces a cascade of stressors—violence, displacement, and resource scarcity—which affect eating behaviors (Jacobi et al. 2004). Disrupted access to food fosters cycles of deprivation and overconsumption; however, this often mirrors clinical symptoms of EDs, such as binge eating and restrictive dieting (Polivy and Herman 1985). In conflict zones, individuals may develop maladaptive coping mechanisms to traverse the physiological and emotional challenges of war. For example, during food scarcity, individuals frequently experience heightened ghrelin secretion (a hormonal response that intensifies hunger), which increases the likelihood of binge eating when food becomes accessible (Hagan and Moss 1997). Studies on famine survivors underscore the enduring impact of chronic food insecurity. Many exhibit long‐term patterns of binge eating, food hoarding, and restrictive dieting (Favaro et al. 2000; Keys 1946). These findings are particularly relevant in the Palestinian context: the Israeli military occupation of Palestine has disrupted food systems and forced many into conditions of acute and chronic scarcity (Aoun et al. 2013).

The psychological toll of conflict, including heightened anxiety and trauma, further exacerbates ED risk by impairing emotional regulation; this fosters reliance on disordered eating as a coping mechanism (Brewerton 2007). However, this dynamic is amplified by the erratic availability of food, which fosters a sense of unpredictability and further entrenches maladaptive eating behaviors. Although understanding how these factors interplay in war‐affected regions is critical for developing effective interventions, it remains a complex issue because it involves many variables and individual circumstances.

Palestinians (who have endured decades of the Israeli military occupation) have created a unique sociopolitical landscape, marked by chronic trauma, food insecurity, and widespread psychological distress. Mental health conditions—such as PTSD, anxiety, and depression—are highly prevalent among Palestinians (Mahamid et al. 2025). This is particularly true for women and adolescents, who are often disproportionately affected by violence and displacement (Marie et al., 2020; Thabet et al. 2015). Research demonstrates a strong correlation between these psychological conditions and disordered eating behaviors; individuals frequently use food to manage emotional distress. For instance, binge eating may provide temporary relief from feelings of helplessness. However, restrictive dieting offers a sense of control in an otherwise chaotic environment (Badrasawi and Zidan; Brewerton 2007). Food insecurity further compounds these risks because erratic access to food fosters cycles of binge eating during abundance and restrictive behaviors during scarcity, mirroring patterns observed in other conflict‐affected populations (Aoun et al. 2013; Polivy et al. 1994).

Cultural disruptions also play a role (as communal eating—a cornerstone of Palestinian identity—has been undermined by displacement and resource scarcity), leading to feelings of isolation among those experiencing EDs. (Marie et al., 2020) These findings emphasize the need to explore the multifaceted factors contributing to EDs in Palestine; however, it is crucial to prioritize culturally sensitive interventions that address both psychological and societal dimensions. Although this might seem like a daunting task, it is necessary because the well‐being of the community depends on it.

Women in Palestine face unique vulnerabilities concerning eating disorders (EDs) that arise from the convergence of caregiving responsibilities, societal pressures, and exposure to violence and displacement. Traditional gender roles frequently place women at the center of family care, thereby necessitating their management of scarce resources during conflict (Veronese et al. 2025; Hamamra et al. 2025). This dynamic often compels women to prioritize their families’ needs over their own, resulting in irregular eating habits and heightened psychological stress (Aoun et al. 2013; Musaiger et al. 2013). Cultural norms further exacerbate the risk of EDs, as women grapple with the demands of adhering to traditional roles while also contending with contemporary beauty standards propagated by global media (Wiederman and Pryor 2000). Adolescents and young women are particularly vulnerable; their exposure to trauma related to conflict during critical developmental stages amplifies the risk of anxiety‐induced eating disorders, including binge eating and anorexia nervosa (Thabet et al. 2015; Kira et al. 2013). These concerning behaviors often persist into adulthood, illustrating the enduring impact of trauma on eating habits (Marie et al. 2016). However, the complexity of these issues cannot be understated.

Addressing the issue requires nuanced understanding of both cultural and psychological factors; it emphasizes interventions that are culturally sensitive, informed by complexities of the Palestinian context (Brewerton 2007; Aoun et al. 2013). However, this understanding is crucial because it shapes effective strategies, although challenges persist.

Although research on men and EDs in Palestine is limited, societal expectations for stoicism and resilience often mask underlying psychological distress; this prevents men from seeking help for disordered eating behaviors (Hoge et al. 2004; Jacobson et al. 2009). However, this highlights the need for inclusive approaches that address gendered experiences of EDs, providing tailored support for both women and men affected by conflict.

The long‐term impact of conflict on eating behaviors is profound; trauma‐induced alterations in eating patterns often persist long after hostilities cease. Prolonged exposure to food insecurity, displacement, and psychological stress creates an environment in which disordered eating behaviors become entrenched (Favaro et al. 2000; Keys 1946). In Palestine, these effects are compounded by intergenerational trauma. Children of war survivors frequently exhibit anxiety, depression, and eating disorder symptoms (Thabet et al. 2015; Kira et al. 2013). Research on famine survivors and displaced populations demonstrates that chronic hunger, coupled with resource deprivation, induces hormonal and metabolic changes that disrupt appetite regulation; this contributes to long‐term patterns of binge eating, food hoarding, and restrictive dieting (Hagan and Moss 1997; Polivy et al. 1994). Cultural disruptions also play a role because the loss of traditional food systems and communal eating practices fosters feelings of isolation and disconnection, further exacerbating eating disorder risk (Marie et al. 2020).

Gendered dynamics intensify these effects, with women often reporting continued struggles with restrictive eating behaviors because of caregiving pressures. Men's ED‐related challenges, however, remain largely unaddressed due to societal stigmas (Aoun et al. 2013; Jacobson et al. 2009). These findings underscore the need for longitudinal studies—to capture the enduring effects of conflict on eating behaviors—and to develop interventions that address both immediate and long‐term needs in war‐affected populations.

Addressing eating disorders in conflict zones requires a multifaceted approach that integrates mental health care, nutritional support, and community‐based interventions. Trauma‐informed care, which prioritizes understanding the psychological impact of conflict, has shown promise in addressing the widespread distress that underpins EDs (Thabet et al. 2015; Kira et al. 2013). For example, cognitive‐behavioral therapy (CBT) tailored to conflict‐affected populations can effectively address anxiety and depression, which are strongly associated with disordered eating behaviors (Favaro et al. 2000; Brewerton 2007). Culturally sensitive approaches are essential; interventions that fail to account for local norms may inadvertently perpetuate stigma or alienate affected individuals (Marie et al. 2016). Educational campaigns that challenge harmful societal norms around body image and mental health—while promoting awareness of EDs as medical conditions—can reduce stigma and encourage help‐seeking behaviors (Musaiger et al. 2013). However, this complex interplay of factors necessitates careful consideration and adaptation to the unique contexts in which these disorders manifest.

Community‐based programs that integrate mental health services into existing healthcare systems can provide accessible support, particularly in regions where resources are scarce. Schools and universities offer opportunities for early intervention with programs focused on stress management, healthy coping strategies, and nutritional education—this proves effective in mitigating ED risks among adolescents and young adults. However, these initiatives, combined with sustained research efforts, are crucial for addressing the complex and multifaceted nature of EDs in conflict‐affected regions, although challenges persist.

### 1.1 Current Study

The current study sought to test the association between traumatic events and EDs and the moderating effect of chosen sociodemographic factors (educational level, gender, region, and age) in the Palestinian context. Based on previous research (Badrasawi and Zidan; Brewerton 2007; Thabet et al. 2015; Kira et al. 2013), the current study hypothesizes that: First, Traumatic events would be positively associated with eating EDs among Palestinians (H1). Second, educational level, gender, region of residency, and age would predict EDs among Palestinians (H2). Third, traumatic events and demographic variables (educational level, gender, region of residency, and age) would predict together EDs among Palestinians (H3).

## 2 Methodology

### 2.1 Participants

We conducted our study in December 2024 and targeted Palestinians in the West Bank, Gaza Strip, and East Jerusalem during a challenging period of political conflict between Israelis and Palestinians. This period witnessed many violent incursions and confrontations between the Israeli soldiers and the Palestinians. Participants of our study were 580, including 320 males and 260 females. The majority, 69.5%, of participants were from urban regions, 17.6 percent were from rural regions, and 12.9 percent were from Palestinian internally displaced camps. A total of 26.1% of participants held a master's degree, 58.4% held a bachelor's degree, and 13.5% held a high school degree. Regarding geographical region, 47.4% participants were from the West Bank, 38.5% from the Gaza strip, and 14.1% from East Jerusalem. participants aged 17–26, 32.5 aged 27–36, 29.4 aged 37–46, 21.5 aged 47–56, and 16.6 aged more than 56 years.

Participants were recruited using online methods; online advertisements, e‐mail campaigns, and social media. The sample size for this study was calculated based on a 95% CI and a 5% margin of error by using the Raosoft software sample size calculator. Based on that, the recommended sample was 580 participants. Inclusion in the study required participants to be 1) Palestinian, 2) living in the West Bank of Palestine, Gaza strip, and East Jerusalem, 3) native Arabic speakers, and 4) free from having any neurodevelopmental and mental disorders. We excluded from our study all participants who do not live in the West Bank of Palestine, the Gaza strip, and East Jerusalem.

### 2.2 Measures

Following standard methodological recommendations for developing our questionnaires (Hambleton et al., 2005), all items were translated and back‐translated from the original English version to Arabic and pilot‐tested by a panel of ten Arab professionals recognized as experts in psychology, counseling, and social work. These professionals evaluated the clarity and relevance of the questions and translation. After completion of the translated draft, the questionnaires were back‐translated into English by an independent expert English editor. The translated version was then pilot tested among 70 participants, separate from the study sample, to assess the content and construct validity of the scales. All measures demonstrated high levels of content and construct validity.

#### 2.2.1 Brief Trauma Questionnaire (BTQ)

The BTQ (Schnurr et al., 1999) is a 10‐item self‐report measure used to determine if responders have ever experienced a traumatic event. This measure assesses inquiry about events, such as experiencing combat, a motor vehicle accident, or the sudden death of a close friend or family member. In either case, exposure to an event should be scored as positive if a respondent says yes to either. Reliability analysis of BTQ indicated a high degree of reliability in evaluating traumatic events of Palestinians (*α* = 0.88).

#### 2.2.2 The Eating Disorder Examination Questionnaire (EDE‐Q)

The EDE‐Q is a 28‐item self‐report scale designed by Fairburn (2008) to test eating disordered behavior. The EDE‐Q includes four subscales: restraint, eating concern, shape concern, and weight concern. The EDE‐Q is a 7‐point Likert scale offering seven different options for the respondents (0–6), with a higher score indicating a higher degree of an eating disorder. A total score EDE‐Q is the sum of the four subscale scores divided by the number of subscales (i.e., four). In our study, we tested the reliability of the scale using Cronbach's alpha for internal consistency; results of reliability indicated high internal consistency of the total scale in the Palestinian context (0.90).

### 2.3 Procedures

The research was conducted in December 2024 and targeted Palestinians in the West Bank of Palestine. The sample was recruited online using convenience sampling techniques. All participants were provided information to decide whether they wanted to participate in the research. They were provided with descriptions of the scales and the purpose of the research. Participants who agreed to participate in the research signed informed consent. The research was conducted in line with the ethical guidelines of the American Psychological Association (APA, 2010) and the Declaration of Helsinki (World Medical Association, 2013) and had been approved by the An‐Najah National University IRB (Approval number: Ref: Med. Nov 2024/20).

### 2.4 Data Analysis

We used descriptive statistics, means, standard deviations, range, skewness, kurtosis, and reliability for our study variables (traumatic events, restraint eating, eating concerns, weight concerns, shape concern and EDs total). In addition, a Pearson Correlation Coefficient between traumatic events, restraint eating, eating concerns, weight concerns, shape concern and EDs total was conducted to evaluate whether there is statistical evidence for a linear relationship among our study variables. Finally, we used hierarchical regression analysis to predict restraint eating, eating concerns, weight concerns, shape concern, and EDs total through demographic variables (educational level, gender, region of residence, and age) in step1. Moreover, demographic variables (educational level, gender, region, and age) with the traumatic events were used to predict restraint eating, eating concerns, weight concerns, shape concern, and EDs total in step2. The hierarchical regression analysis has been tested using SPSS 29 software for data analysis.

## 3 Results

Descriptive statistics related to the traumatic events, restraint eating, eating concerns, weight concerns, shape concern, and EDs total were calculated as shown in Table 1. Participants reported high scores on traumatic events, restraint eating, weight concerns, and EDs and moderate scores on eating concerns, and shape concerns. Regarding the normality of our data, the results of the Kolmogorov‐Smirnov test, along with acceptable levels of skewness and kurtosis, indicated that our data follows a normal distribution. These findings support the assumption of normality and suggest that the use of parametric statistical methods is appropriate. Concerning internal consistency, our scales indicated a high level of reliability on Cronbach's Alpha Formula; scores ranged from 0.91 (*traumatic events*) to 0.91 (*eating concern*).

**TABLE 1:** Descriptive statistics for research variables (N = 580).

Variable	Mean	S.D	Min	Max	Range	Skewness	Kurtosis	Reliability
Traumatic events	1.52	0.21	1.00	2.00	1.00	0.07	−0.82	0.91
Restraint eating	3.60	0.61	1.05	4.98	4.19	0.82	0.23	0.93
Eating concerns	3.40	0.66	1.44	4.85	4.15	0.83	0.108	0.94
Weight concerns	3.60	0.68	1.75	5.00	4.13	0.75	0.33	0.91
Shape concern	3.42	0.64	1.23	4.38	4.33	0.94	0.29	0.92
EDs total	3.58	0.60	1.95	4.32	3.72	0.91	0.37	0.93

Results of the correlational analysis are mentioned in Table 2. Specifically, traumatic events positively correlated restraint eating (*r* = 0.41*, p *< 0.01), eating concern (*r* = 0.42, *p* < 0.01), weight concern (*r* = 0.43, *p *< 0.01), shape concern (*r* = 0.46, *p *< 0.01), and ED total (*r* = 0.40, *p *< 0.01). Restraint eating was positively correlated with eating concern (*r* = 0.71, *p *< 0.01), weight concern (*r* = 0.74, *p *< 0.01), shape concern (*r* = 0.69, *p < 0.01*), and EDs total (*r *= 0.73, *p *< 0.01). Eating concern positively correlated with weight concern (*r* = 0.68, *p *< 0.01), shape concern (*r* = 0.64, *p < 0.01*), and EDs total (*r* = 0.71, *p < 0.01*). Weight concern was positively correlated with shape concern (*r* = 0.69, *p < 0.01*), and EDs total (*r* = 0.68, *p < *0.01). Finally, shape concern was positively correlated with EDs (*r* = 0.64, *p* < 0.01). The results of the correlation analysis showed that traumatic events were positively and significantly correlated with all EDs subscales.

**TABLE 2:** Correlations among study variables (*N* = 580).

Measures	*1*	*2*	*3*	*4*	*5*	*6*
Traumatic events	1	0.41^**^	0.42^**^	0.43^**^	0.46^**^	0.40^**^
Restraint eating		1	0.71^**^	0.74^**^	0.69^**^.	0.73^**^
Eating concerns			1	0.68^**^	0.64^**^	0.71^**^
Weight concerns				1	0.69^**^	0.68^**^
Shape concern					1	0.64^**^
EDs total						1

** Correlation is significant at the 0.01 level (2‐tailed).

In Table 3, we tested hierarchical regression analysis to predict restraint eating, eating concerns, weight concerns, shape concern, and EDs total through demographic variables (educational level, gender, region, and age) in step1. While demographic variables (educational level, gender, region, and age) with traumatic events were used to predict restraint eating, eating concerns, weight concerns, shape concern and EDs total in step2. Our findings revealed that restraint eating was predicted by educational level (*β* = 0.17; ** *p < 0.01*), gender, (*β* = 0.09; ** *p < 0.05*), region (*β* = 0.10; ** *p < 0.05*), age (*β* = ‐0.17; ** *p < 0.01*), and traumatic events (*β* = 0.41; ** p < 0.01). In addition eating concerns predicted by educational level (*β* = 0.16; ** *p   0.01*), gender, (*β* = 0.07; ** *p < 0.05*), region (*β* = 0.13; ** *p < 0.05*), age (*β* = ‐0.14; ** *p < 0.01*), and traumatic events (*β* = 0.42; ** p < 0.01). Weight concerns were predicted by educational level (*β* = 0.18; ** *p < 0.01*), gender, (*β* = 0.09; ** *p < 0.05*), region (*β* = 0.10; ** *p < 0.05*), age (*β* = ‐0.15; ** *p < 0.01*), and traumatic events (*β* = 0.46; ** p < 0.01). Moreover, shape concerns were predicted by educational level (*β* = 0.18; ** *p < 0.01*), gender, (*β* = 0.10; ** *p < 0.05*), region (*β* = 0.10; ** *p < 0.05*), age (*β* = ‐0.16; ** *p < 0.01*), and traumatic events (β = 0.38; ** p < 0.01). Lastly, EDs are predicted to be total by educational level (*β* = 0.16; ** *p < 0.01*), gender, (*β* = 0.10; ** *p < 0.05*), region (*β* = 0.09; ** *p < 0.05*), age (*β* = ‐0.16; ** *p < 0.01*), and traumatic events (*β* = 0.44; ** p < 0.01). As mentioned earlier, all R^2^ values for traumatic events in predicting eating disorders were significant (*p ≤ 0.01*), indicating that traumatic events positively predicted eating disorders.

**TABLE 3:** Hierarchical regression analysis for variables predicting ED (N = 580).

Variable	B	SEB	*Β*	R2
*Step1*		***Restraint eating***		0.11
Educational level	0.16	0.02	0.17^**^	
Gender	0.11	0.04	0.09^*^	
Region of residence	0.10	0.04	0.10^*^	
Age	−0.11	0.02	−0.17^**^	
*Step2*				
Educational level	0.09	0.04	0.10^**^	0.26
Gender	0.08	0.04	0.11^*^	
Region of residence	0.03	0.03	0.09^*^	
Age	−0.13	0.09	−0.21^**^	
Traumatic events	0.42	0.02	0.41^**^	
		***Eating concerns***		
*Step1*				
Educational level	0.14	0.03	0.16^**^	0.10
Gender	0.09	0.04	0.07^*^	
Region of residence	0.17	0.05	0.13^**^	
Age	−0.08	0.02	−0.14^**^	
*Step2*				
Educational level	0.09	0.03	0.10^**^	0.24
Gender	0.06	0.03	0.08^*^	
Region of residence	0.09	0.04	0.09^*^	
Age	−0.09	0.01	−0.13^**^	
Traumatic events	0.45	0.02	0.42^**^	
		***Weight concerns***		
*Step1*				
Educational level	0.16	0.03	0.18^**^	0.12
Gender	0.144	0.047	0.095^**^	
Region of residence	0.145	0.043	0.104^**^	
Age	−0.102	0.021	−0.158**	
*Step2*				
Educational level	0.13	0.03	0.12^**^	0.29
Gender	0.09	0.05	0.09^*^	
Region of residence	0.07	0.03	0.06^*^	
Age	−0.11	0.01	−0.17^**^	
Traumatic events	0.44	0.03	0.46^**^	
		***Shape concern***		
*Step1*				
Educational level	0.17	0.03	0.18^**^	0.10
Gender	0.15	0.04	0.010^**^	
Region of residence	0.14	0.04	0.11^**^	
Age	−0.10	0.02	−0.16^**^	
*Step2*				
Educational level	0.13	0.03	0.13^**^	0.26
Gender	0.11	0.04	0.09^*^	
Region of residence	0.08	0.03	0.09^*^	
Age	−0.07	0.01	−0.19^**^	
Traumatic events	0.39	0.030	0.38^**^	
		***EDs total***		
*Step1*				
Educational level	0.18	0.03	0.16^**^	0.11
Gender	0.14	0.04	0.10^**^	
Region of residence	0.14	0.03	0.09^**^	
Age	−0.12	0.02	−0.16^**^	
*Step2*				
Educational level	0.17	0.04	0.12^**^	0.27
Gender	0.13	0.03	0.09^*^	
Region of residence	0.14	0.02	0.08^*^	
Age	−0.11	0.01	−0.17^**^	
Traumatic events	0.17	0.02	0.44^**^	

** P < 0.01; ^*^p < 0.05.

## 4 Discussion

The current study sought to test the association between traumatic events and eating disorders (restraint eating, eating concerns, weight concerns, shape concern, and EDs total), and the moderating effect of chosen sociodemographic factors (educational level, gender, region, and age) in the Palestinian context.

Consistent with the first study hypothesis, traumatic events were positively correlated with EDs (restraint eating, eating concerns, weight concerns, shape concern, and EDs total) among Palestinian adults, which also supports prior research findings (Brewerton et al. 2020; Convertino and Mendoza 2023; Convertino et al. 2022). This result can be explained by the harsh and extremely difficult living conditions experienced by Palestinians residing in the West Bank, Gaza Strip, and East Jerusalem. These conditions include what Palestinians in Gaza consider a form of genocide, manifested in the complete removal of entire population clusters and the displacement of the majority of Gazans to the Rafah area. Furthermore, Palestinians in the West Bank suffer from extremely difficult conditions due to the ongoing incursions into Palestinian cities, villages, and camps, as well as the continuous arrests of Palestinians at Israeli checkpoints.

The Israeli occupation authorities also adopt highly repressive policies targeting Palestinians in East Jerusalem, including the constant demolition of buildings and attempts to displace the Arab population of the city outside the boundaries of Jerusalem. All of these events contribute to increased psychological stress and traumatic experiences among Palestinians, which directly impact the occurrence of eating disorders. The difficult conditions individuals face can lead to various psychological disturbances, and one of the most significant of these is eating disorders. These disorders manifest in the deterioration of individuals' diets and the development of negative attitudes towards food and body image in general. This includes disorders such as anorexia nervosa, bulimia nervosa, and other eating disorders related to food intake.

In a systematic review study by Convertino and Mendoza (2023), which aimed to systematically review the literature on traumatic events and/or PTSD as either predictors or moderators of EDs, the results indicated that traumatic event exposure was associated with greater EDs prevalence. Convertino et al. (2022) tested the association between traumatic events and EDs in the United States. The results indicated a strong positive association between traumatic events and EDs.

Consistent with the second and third study hypotheses, EDs were found to be predicted by sociodemographic factors and traumatic events, in which educational level, gender, region, and age predicted restraint eating, eating concerns, weight concerns, shape concern, and ED total. These EDs were also predicted by both the sociodemographic factors studied and traumatic events. These findings reflect previous research findings (Burnette et al. 2022; Mitchison and Hay 2014). Furthermore, this can be understood in the context that EDs can be influenced by a combination of sociodemographic factors, along with contextual, environmental, personal, or cultural influences, all of which impact an individual's mental health and mood.

The results of the current study showed that EDs among females in the Palestinian context are higher than those among males. This can be explained by the severe pressures Palestinian women have faced recently, particularly in the wake of the events in Gaza. Palestinian women have shouldered significant family burdens, in addition to playing various roles such as working in different professions, dealing with Israeli military checkpoints, and facing arrests, all while managing family responsibilities.

The results also revealed that individuals with lower educational levels exhibited higher levels of eating disorders compared to those with higher educational qualifications. This can be attributed to the difficult economic pressures faced by the working class in Palestine, especially after the events of October 7, 2023. Unemployment has risen significantly in Palestine, which has increased psychological stress among working individuals in Palestinian society.

Furthermore, the results indicated that residents of Palestinian refugee camps displayed higher levels of eating disorders compared to those living in cities and villages. This can be attributed to the extreme pressures currently faced by refugee camps, including the destruction of infrastructure and the ongoing arrests of camp residents, which have reached a level higher than that experienced by those in Palestinian cities and villages.

Regarding age, the study found that younger individuals experienced higher levels of eating disorders compared to older individuals. This can be associated with the nature of the events currently affecting Palestinian youth, particularly in Gaza. The Palestinian youth, especially in Gaza, have been exposed to an overwhelming number of traumatic events, such as the destruction of educational institutions, arrests, and the injuries and deaths of thousands, which likely contribute to an increase in psychological disturbances among this group in general, and eating disorders specifically.

Compared to previous studies, our research offers novel insights for both researchers and mental health practitioners. This is largely due to the distinctive characteristics of Palestinian society, where individuals have faced continuous traumatic events for over 76 years. Unlike earlier studies, this one is unique in that it includes a diverse range of groups within Palestinian society, such as those living in Palestinian camps. These groups have experienced specific and prolonged hardships, which are often underrepresented in the literature. The findings of this study are particularly significant for mental health providers in Palestine. By understanding the impact of long‐term trauma on various subgroups, mental health professionals will be better equipped to design interventions that address the specific needs of these populations. These interventions will focus not only on alleviating the effects of trauma but also on treating related disorders, such as EDs, which have been shown to be linked to ongoing traumatic experiences.

The results also provide an opportunity for mental health providers to develop culturally sensitive and contextually appropriate treatments. By combining international best practices in psychotherapy with culturally‐based strategies tailored to Palestinian society, mental health practitioners can ensure their approaches are both effective and respectful of local traditions. This dual approach will enhance the overall well‐being of individuals affected by trauma and contribute to the long‐term mental health resilience of Palestinian communities.

### 4.1 Limitations

Several limitations to our study must be noted. The results are limited to specific sociodemographic factors. First, the measures used in this study are based on self‐reports with subjective responses without ties to specific behavioral indicators, which may increase the effect of positive self‐presentation. Self‐report measures often inflate scores of traumatic events and decrease scores of eating disorders depending on the cultural norms of the population. Future research could incorporate multi‐method approaches, combining self‐reports with objective behavioral measures and clinical interviews to provide a more accurate assessment. Second, this study was conducted using a quantitative methodology and solely relied on questionnaires and self‐reports completed by the participants. It must be remembered that self‐reported data may reflect no more than a tendency. It is therefore recommended to use mixed‐method tools in future studies. Third, one limitation of the study is the potential influence of demographic variables, such as geographic location (urban vs. rural), on the reporting of traumatic events and eating disorders. Differences in access to healthcare, social norms, and cultural attitudes between urban and rural populations may lead to variations in how participants perceive, report, or even experience trauma and eating‐related issues. Future studies should account for demographic factors such as geographic location and employ stratified sampling to ensure equal representation of urban and rural populations, considering differences in healthcare access, social norms, and cultural perspectives.

## 5 Conclusion

The current study emphasized the critical impact of traumatic events and sociodemographic factors on EDs among Palestinians. The findings demonstrated that traumatic events significantly contribute to explaining the variance in EDs. Our results also highlighted the moderating role of specific sociodemographic factors, such as educational level, gender, region, and age, in explaining the variance in EDs. Supporting Palestinians in coping with traumatic events could, in some way, reduce EDs, thus improving mental health and well‐being outcomes within the Palestinian population.

The study underscores the positive impact of mental health services in enhancing quality of life and addressing mental health challenges in Palestinian society, which is marked by a high prevalence of traumatic events and ongoing political conflict. Finally, the findings of the current study open the door for further research to explore how the variables in this study are interconnected. This deeper understanding could lead to the development and implementation of effective clinical interventions aimed at reducing EDs by promoting positive coping strategies for dealing with traumatic events, with the support of mental health professionals. Future research could also focus on conducting experimental and longitudinal studies to examine the long‐term effects of traumatic events on mental well‐being among Palestinians. Such studies would provide valuable insights that could help Palestinian authorities, represented by the Ministry of Education, the Ministry of Health, and non‐governmental organizations, to develop comprehensive prevention programs and therapeutic techniques tailored to the unique needs of Palestinian communities. These efforts would contribute to enhancing mental health support and improving overall well‐being in Palestine.

## Author Contributions

All authors contributed equally to this work. All authors read and approved the final manuscript.

## Ethics Statement

All procedures performed in this study involving human participants were in accordance with the ethical standards of An‐Najah University Research Ethics Board (IRB), the American Psychological Association (APA, 2010) and with the Helsinki Declaration (World Medical Association, 2013). Informed consent was obtained from all participants. The protocol of our study was received ethical approval from An‐ Najah National University Research Ethics Board (IRB) before data collection was initiated.

## Consent

The authors have nothing to report.

## Conflicts of Interest

The authors declare no conflict of interests.

### Peer Review

The peer review history for this article is available at https://publons.com/publon/10.1002/brb3.70460

## Data Availability

The datasets generated during and/or analyzed during the current study are available from the corresponding author on request.
